# Protocol to evaluate the efficacy and safety of tolvaptan in patients with refractory ascites after liver resection: an open-label, single-arm phase I/II study

**DOI:** 10.1097/SP9.0000000000000015

**Published:** 2023-12-15

**Authors:** Yosuke Namba, Tsuyoshi Kobayashi, Shintaro Kuroda, Masakazu Hashimoto, Daisuke Takei, Sotaro Fukuhara, Ko Oshita, Keiso Matsubara, Naruhiko Honmyo, Ryosuke Nakano, Hiroshi Sakai, Hiroyuki Tahara, Masahiro Ohira, Kentaro Ide, Hideki Ohdan

**Affiliations:** a Department of Gastroenterological and Transplant Surgery Applied Life Sciences, Institute of Biomedical and Health Sciences, Hiroshima University; b Department of Gastroenterological-Breast and Transplant Surgery, Hiroshima Prefectural Hospital; c Department of Surgery and Endoscopic Surgery, JA Onomichi General Hospital, Hiroshima, Japan

**Keywords:** hepatic resection, postoperative ascites, refractory ascites, tolvaptan

## Abstract

**Background:**

In patients with chronic liver diseases such as cirrhosis, massive ascites after hepatic resection is the cause of prolonged hospitalization and worsening prognosis. Recently, the efficacy of tolvaptan in refractory ascites has been reported; however, there are no reports on the efficacy or safety of tolvaptan for refractory ascites after hepatic resection. This study aims to evaluate the efficacy of early administration of tolvaptan in patients with refractory ascites after hepatic resection.

**Materials and methods:**

This is an open-label, single-arm phase I/II study. This study subject will comprise patients scheduled for hepatic resection of a liver tumor. Patients with refractory ascites after hepatic resection (drainage volume on postoperative day 1 ≥5 ml/body weight 1 kg/day) will be treated with tolvaptan. The primary endpoint will include the maximum change in body weight after hepatic resection relative to the preoperative baseline. The secondary endpoints will include drainage volume, abdominal circumference, urine output, postoperative complication rate (heart failure and respiratory failure), number of days required for postoperative weight gain because of ascites to decrease to preoperative weight, change in improvement of postoperative pleural effusion, total amount of albumin or fresh frozen plasma transfusion, type and amount of diuretics used, and postoperative hospitalization days.

**Conclusion:**

This trial will evaluate the efficacy and safety of tolvaptan prophylaxis for refractory ascites after hepatic resection. As there are no reports demonstrating the efficacy of tolvaptan prophylaxis for refractory ascites after hepatic resection, the authors expect that these findings will lead to future phase III trials and provide valuable indications for the selection of treatments for refractory postoperative ascites.

## Introduction

Highlights

- Massive ascites after hepatic resection is the cause of prolonged hospitalization and worsening prognosis.
- No study has reported on the efficacy or safety of tolvaptan for refractory ascites after hepatic resection.
- This trial will evaluate the efficacy and safety of tolvaptan prophylaxis for refractory ascites after hepatic resection.
- These findings will lead to future phase III trials and provide valuable indications for the selection of treatments for refractory postoperative ascites.

Postoperative ascites is one of the most common complications after liver resection and is reported in 5–56% of patients with liver resection^1,2^. Despite advancements in perioperative management and patient selection, the incidence rate of postoperative ascites remains high. The reduction in functional liver tissue due to hepatic resection leads to postoperative sepsis, nutritional deprivation, and compromised immunity, which further aggravates liver function^3^. In addition, as several patients have chronic liver diseases, such as cirrhosis, postoperative deterioration of liver function and portal hypertension due to intraoperative manipulation can lead to a large accumulation of ascites^4–6^. Postoperative ascites accumulation requires prolonged hospitalization after hepatic resection due to prolonged drainage and infection. The continuous loss of large amounts of plasma in ascites can also lead to liver failure^7–9^, and the occurrence of postoperative ascites after liver resection increases the risk of long-term mortality^10^. Furthermore, patients with refractory ascites after liver resection are more expensive to treat due to prolonged hospitalization and albumin administration^8,11^.

Based on the clinical practice guidelines for liver cirrhosis published by the Japanese Society of Gastroenterology, spironolactone is the first choice for patients with frequent drainage after hepatic resection, and furosemide is used when the effect of spironolactone is unsatisfactory^12^. However, spironolactone is refractory in ~15–20% of patients with ascites and does not respond to diuretic therapies^13^. Additionally, the use of high doses of spironolactone causes renal function deterioration and mineral abnormalities^14^. Patients with refractory ascites can be treated with various therapies, including concentrated ascites reinfusion therapy, peritoneovenous shunting, and transjugular intrahepatic portosystemic shunting; however, their efficacy is limited^15–17^.

Recently, the efficacy of the additional use of tolvaptan in patients with cirrhosis has been demonstrated. In patients who do not respond to existing diuretic therapy, tolvaptan results in weight loss, ascites reduction, increased urine output, and improved symptoms related to edema in ~60% of patients^18–21^. Several studies have reported that tolvaptan use in patients with refractory ascites improves their long-term prognosis^22,23^. Furthermore, tolvaptan results in significantly lower renal dysfunction in patients with ascites effusions due to cirrhosis treated with tolvaptan compared with those not treated with tolvaptan^24,25^.

Tolvaptan is an orally selective vasopressin type 2 receptor antagonist that inhibits water reabsorption by decreasing aquaporin 2 expression in the collecting duct, thereby promoting free water excretion without increasing sodium excretion^26,27^. It has also been reported to maintain renal blood flow during diuresis without activating the renin-angiotensin-aldosterone system^28,29^. As tolvaptan can improve congestive symptoms without impairing renal function, its efficacy in patients with acute heart failure and complications after cardiac surgery is also attracting attention^30,31^.

For these reasons, we hypothesized that the prophylactic use of tolvaptan in patients with refractory ascites after hepatic resection would be effective in controlling ascites and protecting renal function. However, there are no reports regarding the efficacy of tolvaptan in patients with refractory postoperative ascites.

This study aims to determine whether tolvaptan can be used for the early control of refractory postoperative ascites. To the best of our knowledge, this is the first study to investigate the safety and efficacy of early tolvaptan in refractory ascites after hepatic resection, and valuable indications for the selection of treatments for refractory postoperative ascites.

## Methods/design

### Study design

**Figure 1:**
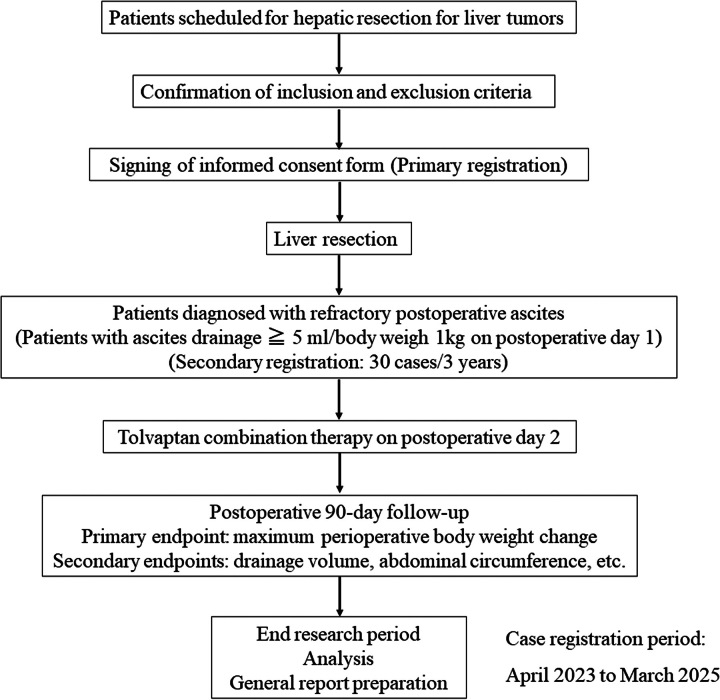
Study design.

This single center, open-label, single-arm phase I/II clinical study will evaluate the efficacy and safety of early ascites control using tolvaptan in patients with ascites effusion after hepatic resection. The primary endpoint of this study will be the maximum perioperative weight change. The study design is illustrated in Figure 1. The case registration period for this study is from March 2023 to December 2025. The study protocol was issued on 21 December 2022 and the amendment number is 1.0. This study protocol was approved by the Institutional Review Board of Hiroshima University, Japan (approval no. CRB2022-0018).

### Study objectives and endpoints

The primary endpoint of this study will be the maximum change in body weight (BW) after hepatic resection relative to the preoperative baseline. We consider using drainage volume as the primary endpoint; however, drainage volume is highly variable and inaccurate. Weight change correlates well with drainage volume and has less variability; hence, we use weight change as the primary endpoint. The secondary endpoints will be the following: (1) drainage volume, (2) abdominal circumference, (3) urine output, (4) postoperative complication rate (heart failure and respiratory failure), (5) number of days required for postoperative weight gain because of ascites to decrease to preoperative weight, (6) change in improvement of postoperative pleural effusion, (7) total amount of albumin or fresh frozen plasm (FFP) transfusion, (8) type and amount of diuretics used, and (9) postoperative hospitalization days.

### Patient selection and enrollment criteria

#### Target disease

Liver tumors (primary or metastatic liver cancer)

#### Inclusion criteria

1. Primary registration
  - Scheduled for hepatic resection for liver tumors (primary or metastatic liver cancer);
  - Aged ≥20 years at the time of consent;
  - Performance status score of 0 or 1;
  - White blood cells ≥2000/mm^3^, hemoglobin ≥7.0 g/dl, platelets ≥30 000/mm^3^, total bilirubin ≤5.0 mg/dl, and creatinine ≤2.0 mg/l;
  - Written informed consent.
2. Secondary registration
  - Drainage volume of ≥5 ml/BW 1 kg/day on postoperative day 1.

#### Exclusion criteria

1. Primary registration
  - Preoperatively taking tolvaptan;
  - History of serious drug hypersensitivity or hypersensitivity to any component of tolvaptan or similar compounds;
  - Difficulty with fluid intake or adequate hydration;
  - Pregnant or may become pregnant;
  - Severe ischemic heart disease or severe valvular heart disease (New York Heart Association classification ≥III);
  - Severe hepatic cirrhosis (liver damage C);
  - Renal dysfunction (creatinine ≥2.0) and hyperkalemia (potassium > 5.5 mEq/l);
  - Dyspnea requiring oxygen administration due to severe respiratory impairment (Hugh–Jones classification ≥IV), interstitial pneumonia, or pulmonary fibrosis;
  - Serious preoperative complications, such as infection or gastrointestinal bleeding;
  - Requiring gastrointestinal anastomosis, biliary reconstruction, lymph node dissection, revascularization, or complicated resection of other organs other than the gallbladder;
  - Psychosis or psychiatric symptoms that make study participation difficult.
2. Secondary registration
  - Difficulty with fluid intake or adequate hydration;
  - Serious postoperative complications, such as infection or gastrointestinal bleeding;
  - Performed gastrointestinal anastomosis, biliary reconstruction, lymph node dissection, revascularization, or complicated resection of other organs other than the gallbladder.

### Sample size calculation

In a phase III clinical trial conducted by Sakaida *et al.* ^18^ on the efficacy of tolvaptan in patients with cirrhosis with hepatic edema, weight losses were –0.44 kg (SD, 1.93) in the placebo group (*n*=80) and –1.95 kg (SD, 1.77) in the tolvaptan group (*n*=82). In a dose-ranging study of tolvaptan in patients with cirrhosis with hepatic edema conducted by Okita *et al*.^19^ weight losses were –0.36 kg (SD, 2.06) in the placebo group (n=25) and –2.3 kg (SD, 2.35) in the tolvaptan group (n=25). In other words, 1–2 kg of weight loss is expected with tolvaptan.

In our previous data, a postoperative weight gain of ~3 kg was observed patients with refractory ascites who underwent drainage of 5 ml/BW 1 kg on the first day after hepatectomy. As the same weight and ascites loss as in previous literature are expected to occur, it is assumed that the use of tolvaptan can limit postoperative weight gain to 2 kg. Assuming an SD of 2.0, an effect size of 0.5, a power of 0.9, and a significance level of 0.1, 25 cases are required; considering that the dropout rate is ~10%, 30 cases are set as the required number of cases.

### Interventions

#### Tolvaptan oral administration

For patients with ascites drainage ≥5 ml/BW 1 kg on postoperative day 1, tolvaptan 7.5 mg will be added to potassium canrenoate 200 mg (or spironolactone 50 mg) starting on postoperative day 2. Tolvaptan will be administered once daily in a glass of water. When a patient forgets to take a dose of tolvaptan, the patient will take tolvaptan immediately if it is within 4 h of the scheduled dose; if ≥4 h has passed, the patient will resume taking the drug as usual the next time. The dose may be reduced or discontinued if possible as the disease improves.

#### Concomitant diuretics

Potassium canrenoate 200 mg intravenously once daily or spironolactone 50 mg orally once daily will be the basic treatment on postoperative day 1. Similar to tolvaptan, potassium canrenoate can be reduced or discontinued as required as symptoms improve. If the ascites is difficult to control, potassium canrenolate or hurosemide may be added. Other concomitant therapies commonly used in daily practice are not restricted. The treatment duration for refractory ascites, including oral tolvaptan, should range from 2 weeks to 1 month.

### Follow-up and assessment of efficacy

#### Assessment tools

The maximum perioperative weight change will be the primary endpoint of this study. The secondary endpoints will include drainage volume, abdominal circumference, and urine output. Blood tests, chest radiography, and computed tomography (CT) will be used to evaluate the incidence of postoperative complications and changes in the improvement of postoperative pleural effusion. To assess patient safety, blood sodium and potassium levels and liver and renal functions will be evaluated.

#### Observation and examination schedule

Urine output, BW, and abdominal circumference will be measured daily until discharge. Preoperative BW should be measured within 7 days prior to surgery, and BW should be measured as soon as possible after waking up. Drainage volume should be measured daily until the drain is removed. The timing of the drain removal will be at the discretion of the attending physician. Blood tests will be performed daily until 7 days postoperatively, and after 10 days at the discretion of the attending physician. Preoperative blood tests should be performed within 90 days prior to surgery. Chest radiography will be performed within 90 days before surgery, immediately after surgery, and on first, third, fifth, and seventh postoperative days. CT will be performed according to the patient’s condition. After discharge, BW and abdominal circumference measurements, blood tests, and chest radiography will be performed during outpatient visits 30 and 90 days postoperatively. The patients will also be evaluated for adverse events. Observation will be completed 90 days postoperatively.

### Statistical analysis

The primary objective of the study will be to determine the efficacy and safety of tolvaptan therapy. For items observed as continuous values, the number of examples, number of missing examples, mean (median), SD (quartile), and range (minimum-maximum) will be calculated as summary statistics. For items observed as discrete values, the number of examples in each category and their percentages will be calculated as summary statistics. For safety evaluation, the number and percentage of adverse events will be calculated using the subjects for whom at least one research treatment has been performed as the analysis population. Statistical tests for the main analysis will be performed at a 5% significance level (two-sided).

## Discussion

This trial will evaluate the efficacy and safety of tolvaptan prophylaxis for refractory ascites after hepatic resection. The primary endpoint will be perioperative maximum weight change. The secondary endpoints will include drainage volume, abdominal circumference, urine output, postoperative complication rate, number of days required for postoperative weight gain because of ascites to decrease to preoperative weight, change in improvement of postoperative pleural effusion, total amount of albumin or FFP transfusion, type and amount of diuretics used, and postoperative hospitalization days.

Tolvaptan is an orally selective vasopressin type 2 receptor antagonist that inhibits water reabsorption by decreasing aquaporin 2 expression in the collecting duct, thereby promoting free water excretion without increasing sodium excretion^26,27^. It is expected to be renal protective by maintaining renal blood flow during diuresis without activating the renin-angiotensin-aldosterone system^28,29^.

In 2013, a Japanese clinical trial approved the use of tolvaptan in combination with a conventional diuretic for refractory ascites^32^. Subsequently, the efficacy of tolvaptan for refractory ascites was confirmed regardless of serum albumin levels^18^, and the Japanese Society of Gastroenterology recommended early administration of tolvaptan before increasing the dose of furosemide or spironolactone^33^.

Some reports have indicated that tolvaptan improves the long-term prognosis in patients with refractory ascites. Hiramine *et al.* ^22^demonstrated that the survival rate was significantly higher in the tolvaptan using group than in the tolvaptan nonusing group in patients with hepatic ascites. A meta-analysis also demonstrated that tolvaptan significantly improved the overall survival of patients with cirrhosis and refractory ascites^29^. Tolvaptan has been reported to reduce the incidence of acute kidney injury in patients with hepatic ascites^24^. Acute kidney injury has also been reported to be induced by diuretics^34^, and the concomitant use of tolvaptan is important for renal protection. Based on these studies, we hypothesized that early administration of tolvaptan would be effective for the treatment of refractory ascites after hepatic resection.

As there are no reports demonstrating the efficacy of tolvaptan prophylaxis for refractory ascites after hepatic resection, we will analyze the safety and efficacy of this therapy in this clinical trial. We expect that these findings will lead to future phase III trials and provide valuable indications for the selection of treatments for refractory postoperative ascites.

This study protocol has several limitations. Firstly, it is a single-arm study, not a comparative study. Second, some confounding factors cannot be completely ruled out. Postoperative ascites may be influenced by the volume of resected liver, liver damage, and operative time. If positive results are obtained in this phase I/II study, we plan to conduct a phase III study including a control group to control for confounding factors. Within the limitations of this study, we believe that this study to evaluate the efficacy and safety of tolvaptan in patients with refractory ascites after hepatic resection is a useful tool that can lead to a phase III study.

## Ethics approval and consent to participate

The protocol was approved by the Certified Committee for Regenerative Medicine, Hiroshima

University, Japan (CRB2022-0018).

## Consent for publication

Informed consent will be obtained from all patients before inclusion in this study.

## Sources of funding

This work was supported by Japan Agency for Medical Research and Development under Grant Number JP23fk0210108 to Hideki Ohdan. The funders had no role in study design, data collection and analysis, decision to publish, or preparation of the manuscript.

## Author contribution

Y.N.: writing – original draft and data curation; T.K.: project administration, conceptualization, and writing – review and editing; S.K.: methodology, validation, and writing – review and editing; M.H.: formal analysis, investigation, writing – review and editing; D.T.: data curation; S.F., K.O., K.M., N.H., R.N., H.S., H.T., M.O., and K.I.: writing – review and editing; H.O.: supervision and funding acquisition.

## Conflicts of interest disclosure

The authors declare that they have no competing interests in this study.

## Research registration unique identifying number (UIN)

The trial was prospectively registered at Japan Registry of Clinical Trials (jRCTs061220114).

## Guarantor

Tsuyoshi Kobayashi and Hideki Ohdan.

## Availability of data and materials

No datasets were generated or analyzed during the current study. All relevant data from this study will be made available upon study completion.
